# A Radiomics Nomogram for Distinguishing Benign From Malignant Round-Like Breast Tumors

**DOI:** 10.3389/fonc.2022.677803

**Published:** 2022-04-26

**Authors:** Lanyun Wang, Yi Ding, Wenjun Yang, Hao Wang, Jinjiang Shen, Weiyan Liu, Jingjing Xu, Ran Wei, Wenjuan Hu, Yaqiong Ge, Bei Zhang, Bin Song

**Affiliations:** ^1^ Department of Radiology, Minhang Hospital, Fudan University, Shanghai, China; ^2^ Department of General Surgery, Minhang Hospital, Fudan University, Shanghai, China; ^3^ Department of Medical Examination Center, Minhang Hospital, Fudan University, Shanghai, China; ^4^ General Electric (GE) Healthcare, Shanghai, China; ^5^ Department of Radiology, Ruijin Hospital, School of Medicine, Shanghai Jiao Tong University, Shanghai, China

**Keywords:** machine learning, radiomics nomogram, round-like tumors, digital mammography, breast

## Abstract

**Objective:**

The objective of this study is to develop a radiomics nomogram for the presurgical distinction of benign and malignant round-like solid tumors.

**Methods:**

This retrospective trial enrolled patients with round-like tumors who had received preoperative digital mammography (DM) no sooner than 20 days prior to surgery. Breast tumors were segmented manually on DM images in order to extract radiomic features. Four machine learning classification models were constructed, and their corresponding areas under the receiver operating characteristic (ROC) curves (AUCs) for differential tumor diagnosis were calculated. The optimal classifier was then selected for the validation set. After this, predictive machine learning models that employed radiomic features and/or patient features were applied for tumor assessment. The models’ AUC, accuracy, negative (NPV) and positive (PPV) predictive values, sensitivity, and specificity were then derived.

**Results:**

In total 129 cases with benign and malignant tumors confirmed by pathological analysis were enrolled in the study, including 91 and 38 in the training and test sets, respectively. The DM images yielded 1,370 features per patient. For the machine learning models, the Least Absolute Shrinkage and Selection Operator for Gradient Boosting Classifier turned out to be the optimal classifier (AUC=0.87, 95% CI 0.76-0.99), and ROC curves for the radiomics nomogram and the DM-only model were statistically different (*P*<0.001). The radiomics nomogram achieved an AUC of 0.90 (95% CI 0.80-1.00) in the test cohort and was statistically higher than the DM-based model (AUC=0.67, 95% CI 0.51-0.84). The radiomics nomogram was highly efficient in detecting malignancy, with accuracy, sensitivity, specificity, PPV, and NPV in the validation set of 0.868, 0.950, 0.778, 0.826, and 0.933, respectively.

**Conclusions:**

This radiomics nomogram that combines radiomics signatures and clinical characteristics represents a noninvasive, cost-efficient presurgical prediction technique.

## Background

Breast cancer (BC) represents the most common malignant disease in females globally (1–3). However, BC mortality has declined remarkably since the 1970s (4) due in large part to mammography screening and enhanced systemic therapy (5, 6). Digital mammography (DM) constitutes a widely accepted means of breast tumor detection in clinic (6, 7) and has come to play a critical role in the evaluation of breast tumors, taking precedence over other techniques in detecting calcification in breast tumors. Normally, breast cancers display an irregular shape and a spiculated margin in DM images, with or without microcalcification. However, some malignant tumors appear as round-like masses, including mucinous carcinoma, intraductal papillary carcinoma, medullary carcinoma, infiltrating ductal carcinoma, intramammary metastases, metaplastic carcinoma, lymphoma, and phyllodes tumors (8, 9). The margins of these tumors can be circumscribed, microlobulated, and indistinct. Clinicians therefore face a challenge in distinguishing the above tumors from benign lesions by DM alone, especially for dense breast tissues. In addition, the prognosis and clinical treatment of these tumors are necessarily different. Preoperative assessment of round-like tumors can thus help to optimize treatment.

The signals of round-like masses without suspicious malignant or benign macrocalcification in DM are comparable, although their internal structures and densities show substantial differences (9). Previous findings (10) recommend that the classification according to the Breast Imaging Reporting and Data System (BI-RADS) of breast masses found on DM images should be determined in combination with ultrasound (US) or magnetic resonance imaging (MRI), except for completely calcified or fatty masses. Routine imaging techniques such as mammography and US show overt limitations in the differential diagnosis of round-like masses. Although the multimodal technique of breast dynamic contrast-enhanced MRI (DCE-MRI) is highly accurate in distinguishing benign from malignant tumors (11) as well as in differentiating well-circumscribed breast malignant lesions from benign ones (8), it requires contrast media injection and is very expensive. In addition, traditional multimodal diagnosis greatly relies on the radiologist’s experience. Though DM is the most applied technique in assessing breast tumors, no quantitative parameters have yet been derived from DM images (12). Therefore, the identification of a measurable DM marker may greatly increase the diagnostic value of this technique for breast tumors. Radiomics could be used to convert digital images into high-dimensional data by extracting a variety of quantitative indices and could thus help to quantitatively evaluate tumor heterogeneity and improve clinical decision making (13, 14).

To this end, this work aims to develop a radiomics nomogram for distinguishing benign from malignant round-like masses without spiculated margins and suspicious malignant calcification or benign macrocalcification, in order to help optimize treatment plans.

## Methods

### Data Cohort

Our institutional review board approved this retrospective study, with no requirement for informed consent. Individuals who received DM screening with benign (excluding fibroadenoma) or malignant tumors confirmed by pathological analysis were continually enrolled between January, 2017 and December, 2019. Due to a high prevalence of fibroadenoma, patients who received DM screening with fibroadenoma confirmed by pathological analysis were continually enrolled between January and December, 2019. Patient data were obtained from the Picture Archiving and Communication System (PACS) of the Affiliated Minhang Hospital of Fudan University, Shanghai, China.

The inclusion criteria were: (1) the presence of an oval or round tumor; (2) a DM exam carried out within 20 preoperative days, with image quality meeting post-processing requirements; (3) nonmalignant or cancerous breast tumor confirmed by histopathology. The exclusion criteria were: (1) receiving treatments, (chemotherapy, surgery, radiotherapy and/or anti-HER2 therapy) before DM screening; (2) the tumor being incompletely displayed in the cranial caudal (CC) or mediolateral oblique (MLO) views; (3) the tumor being architecturally distorted (except for scarring caused by a previous injury or surgery); (4) the tumor showing calcification of BI-RADS 2/4b/4c/5; (5) the tumor having spiculated margins; and (6) the tumor not being displayed due to extremely dense breasts. Ultimately, 129 masses (51 nonmalignant and 78 cancerous) were included, and their histopathologic diagnoses are presented in **Table 1**. The 129 study cases, age 54.6 ± 13.7 years (range, 23–86 years) old, were randomly assigned to the training (n = 91) and test (n = 38) sets.

**Table 1:** Features of 129 breast tumors confirmed by histology.

Histopathologic type		No. of masses	Proportion (%)	No. of masses with calcifications	BI-RADS category of accompanying calcifications
Benign		51	39.5	0	
	Fibroadenoma	44	34.1	0	
	Intraductal papilloma	2	1.6	0	
	Benign phyllodes tumor	4	3.1	0	
	Tubular gland lymphoma	1	0.7	0	
Malignant		78	60.5	5	
	Invasive ductal carcinoma	53	41.1	4	4a (3)
					3 (1)
	Intraductal papillary carcinoma	8	6.2	0	
	Ductal carcinoma *in situ*	1	0.8	0	
	Neuroendocrine carcinoma	1	0.8	0	
	Malignant phyllodes tumor	3	2.3	0	
	Mucinous carcinoma	11	8.5	1	4a
	Sarcomatoid carcinoma	1	0.8	0	

No., number; BI-RADS, Breast Imaging Reporting and Data System.

### DM and Image Processing

A GE Senographe Essential DM system (GE Healthcare, Milwaukee, WI) was utilized for data acquisition. In every case, optimal MLO and CC view images were converted into Digital Imaging and Communications in Medicine (DICOM) files. ITK-SNAP software (http://www.itk-snap.org) was utilized for breast tumor segmentation, and regions of interest (ROIs) were manually segmented on MLO and CC views independently by two radiologists (WY and LW) with 10 and 14 years of experience in DM image evaluation, respectively. In cases of obscured tumor margins, both radiology experts reached a consensus by performing an additional image analysis.

### Feature Extraction and Selection

Radiomic features were obtained with AK v3.2.2 software (GE healthcare). In total 1,370 features were obtained, including histogram, shape, gray-level co-occurrence matrix (GLCM), gray-level run-length matrix (GLRLM) and gray-level size zone matrix (GLSZM) features. For interobserver agreement evaluation, CC views were randomly chosen in 30 cases, and another radiologist delineated ROIs independently. After this, intraclass correlation coefficients (ICCs) of these features were calculated. Based on the ICC’s 95% confidence interval (CI) (15), values >0.90, from 0.75 to 0.9, from 0.5 to 0.75, and <0.5 were considered to reflect excellent, good, moderate, and poor reliability, respectively. Only features with ICC ≥0.75 were included in subsequent analysis.

The patients were randomized into the training and test sets (ratio of 7:3, respectively). Initially, the maximum correlation minimum redundancy (mRMR) algorithm was used for eliminating redundant and irrelevant parameters in the training set, of which 30 features that showed high correlations with labels were retained. Next, least absolute shrinkage and selection operator (LASSO) analysis with 10-fold cross-validation was performed to further select features *via* λ optimization. The coefficients of select features then underwent compression to zero at the optimal λ value, and only parameters that showed a nonzero coefficient were further retained.

### Patient, DM, and US Features

The following clinical information was obtained from the patients’ medical records: age, sex, family history of breast cancer, life habits (drinking/smoking), and childbearing information. Next, DM data were analyzed by two radiologists, as stated above, who recorded the following parameters: (1) tumor size (maximum diameter); (2) margin (circumscribed, obscured, microlobulated, or indistinct); (3) density (low, equal, or high); and (4) location (depth) (anterior, middle, or posterior). Additionally, US data were recorded as described in the US report. The imaging features of US were: (1) echo pattern (anechoic, hypoechoic, isoechoic, complex cystic, and solid, heterogeneous, or hyperechoic); (2) edge (clear, partially clear, or unclear); (3) shape (regular, partially regular, or irregular); and (4) blood flow (presence or none).

### Radiomics Signature, Clinical Model, and Radiomics Nomogram

Four machine learning models, support vector machine (SVM), k-Nearest Neighbor (k-NN), C-Tree, and logistic regression, were constructed based on the previously obtained optimal feature subset described above. All classifiers underwent training with 10-fold cross-validation with 10 repeats in the training cohort. Their predictive performances were then assessed with cross-validation data and validated in the validation cohort, and the optimal classifier in the validation set was selected. Next, radiomics scores (rad-scores) for various patients were determined. The radiomics signature was evaluated for predictive accuracy by the area under the receiver operating characteristic (ROC) curve (AUC) in both the training and test cohorts.

Clinical data, including age, MG, and US characteristics (continuous data) were analyzed by independent samples *t*-test or the Wilcoxon test (for example, age and tumor size (DM)), and the Chi square test or Fisher’s exact test were carried out for analyzing categorical variables such as tumor size (DM), margin (DM), density (DM), location (depth) (DM), echo pattern (US), edge (US), shape (US), and blood flow (US). Univariate logistic analysis was applied to select risk factors for cancerous tumors (*P*<0.05), and this was followed by backward stepwise multivariate logistic regression and likelihood ratio tests in order to build a clinical prediction model. In order to satisfy the collinearity condition, features with both the largest calculated VIF and VIF >10 were eliminated. The model’s performance was then determined by ROC curve evaluation.

**Figure 1:**
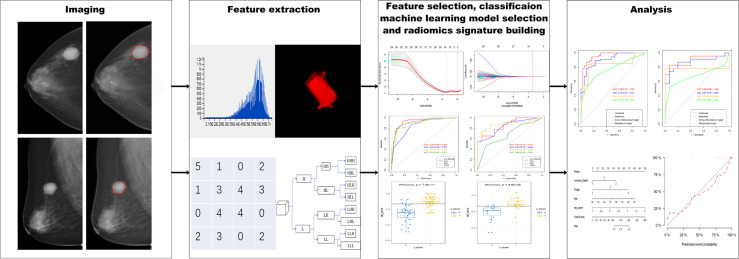
Flow chart of radiomic analysis of round-like masses on DM images.

After this, a radiomics nomogram was built as described above for the clinical model, including the obtained radiomics signature, and its performance was also examined by ROC analysis. Finally, the Hosmer-Lemeshow test was performed to assess consistency between actual and predicted values. The radiomic framework is shown in **Figure 1**.

### Radiomics Nomogram Validation and Evaluation

The radiomics nomogram was examined in the training (n = 91) and test (n = 38) cohorts, respectively, with regard to differentiation, calibration and clinical values, and the AUC was determined in order to evaluate the nomogram’s performance in distinguishing malignant and benign tumors. The Hosmer–Lemeshow test and the calibration curve were utilized as well to determine the goodness-of-fit. Additionally, internal validation was carried out in the test cohort. A rad-score was derived in the test cohort based on the algorithm built in the training cohort, and decision curve analysis (DCA) was performed to estimate the nomogram’s robustness in a clinical setting.

### Statistical Analysis

The software packages *R* v. 3.5.1 (https://www.Rproject.org) and SPSS were utilized for all statistical analysis.

## Results

### Patient and DM/US Features

All patients in this study were female. Regarding some clinical factors, of the 129 patients, three patients (malignant, 2; benign, 1) had a family history of breast cancer; two patients (malignant, 1; benign, 1) had a smoking habit; four patients (malignant, 2; benign, 2) had a drinking habit; and two patients with benign tumors had never given birth. **Table 2** summarizes patient and DM/US features. Age, margin (DM), density (DM), location depth (DM), edge (US), shape (US), and blood flow (US) differed significantly between malignant and benign tumors.

**Table 2:** Patient and DM/US characteristics.

Characteristic		Pathological type		*P* value
		Benign	Malignant	
Margin (DM)	circumscribed	17	18	0.021*
	obscured	31	41	
	microlobulated	0	0	
	indistinct	3	19	
Density (DM)	low-density	1	1	0.000*
	equal-density	41	34	
	high- density	9	43	
Location/Depth (DM)	anterior	7	15	0.050*
	middle	35	38	
	posterior	9	25	
Echo pattern (US)	anechoic	0	1	0.104
	hypoechoic	49	64	
	isoechoic	1	1	
	complex cystic and solid	1	3	
	heterogeneous	0	9	
	hyperechoic	0	0	
Edge (US)	clear	7	3	0.002*
	partially clear	43	58	
	unclear	1	17	
Shape (US)	regular	6	3	<0.001*
	partially regular	44	41	
	irregular	1	34	
Blood flow (US)	none	22	13	0.001*
	presence	29	65	
Age #		45 (41~52)	60.5 (50.5~70)	<0.001*
Size #		1.9 (1.6~2.8)	2.3 (1.6~3.225)	0.158

*means P<0.05; # means nonnormal distribution obtained after SK normality test; DM, digital mammography; US, ultrasound.

In our univariate logistic regression analysis, factors including age, margin (DM), density (DM), location depth (DM), edge (US), shape (US), and blood flow (US) showed significant associations with malignant masses (all *P*<0.05; **Table 3**), and multivariate logistic regression analysis suggested that age, location depth (DM), shape (US), and rad_score were independent predictors of malignant masses (**Table 4**). These clinical variables were then employed to construct a clinical model that had an AUC value of 0.78 (0.61-0.95) in the testing cohort, which was higher than that of DM 0.67 (0.51-0.84).

**Table 3:** Positive results of univariate analysis for the differential diagnosis of round-like breast tumors.

Variable	2.5%CI	97.5%CI	OR value	*P* value
Age	1.031	1.110	1.068	0.001*
Margin (DM)	1.236	3.347	1.951	0.008*
Density (DM)	2.010	13.221	4.917	0.001*
Location_Depth (DM)	1.022	4.422	2.064	0.050
Edge (US)	2.335	51.850	8.197	0.005*
Shape (US)	4.334	95.747	15.082	0.000*
Blood_flow (US)	1.228	9.486	3.321	0.020*

*means P<0.05; DM, digital mammography; US, ultrasound; CI, confidence interval; OR, odds ratio.

**Table 4:** Positive results of multivariate logistic regression analysis for the differential diagnosis of round-like breast tumors.

Variable	2.50%CI	97.50%CI	OR	P value
Location_Depth (DM)	1.197	16.582	3.978	0.036*
Shape (US)	1.900	57.442	7.969	0.013*
Age	1.024	1.134	1.072	0.006*
Rad_score	2.821	33.017	8.060	<0.001*
Intercept	<0.001	<0.001	<0.001	<0.001*

*means P<0.05; DM, digital mammography; US, ultrasound; CI, confidence interval; OR, odds ratio.

### Radiomics for Predictive Modeling

**Figure 2:**
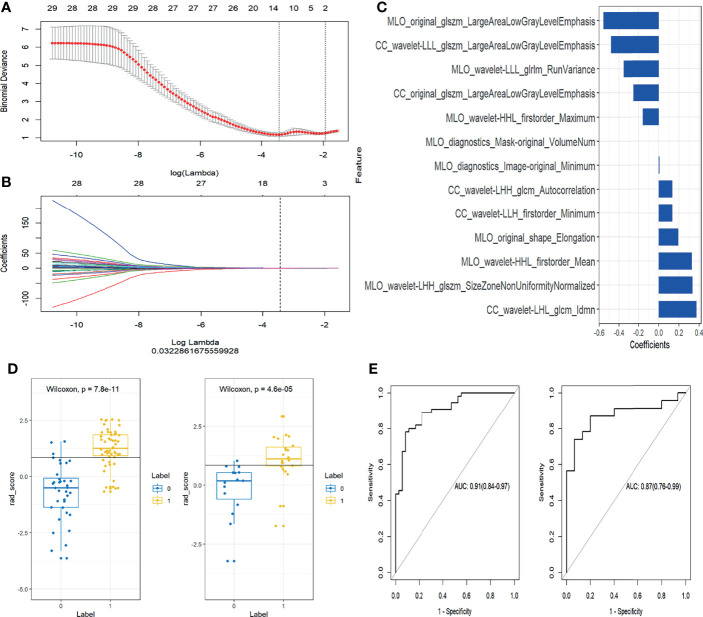
Selection of radiomics features and evaluation of the prediction performance of the radiomics signature. **(A)** Selection of the hyperparameter (λ) in the least absolute shrinkage and selection operator (LASSO) model *via* ten-fold cross-validation based on minimum error; vertical black dotted line, optimal value of λ (best fit). **(B)** Coefficients and log(λ) values; features with nonzero coefficients are shown. **(C)** The 13 features showing nonzero coefficients are displayed. The features utilized for constructing the radiomics signature are shown on the y-axis with the corresponding coefficients in LASSO Cox analysis on the x-axis. **(D)** Rad-scores of benign and malignant masses in the training and test groups. Yellow and blue represent the actual classification: the greater the separation of yellow and blue, the better the rad-score**’**s predictive accuracy. **(E)** Receiver operating characteristic (ROC) curves of the radiomics signature in the training and test set.

**Figure 3:**
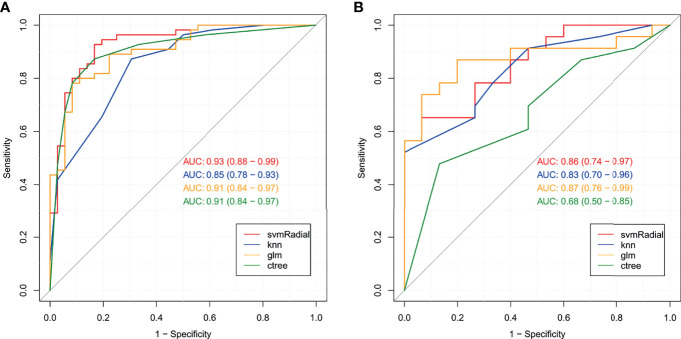
Receiver operating characteristic (ROC) curves for the four classification machine learning models in the training set **(A)** and test set **(B)**.

**Figure 4:**
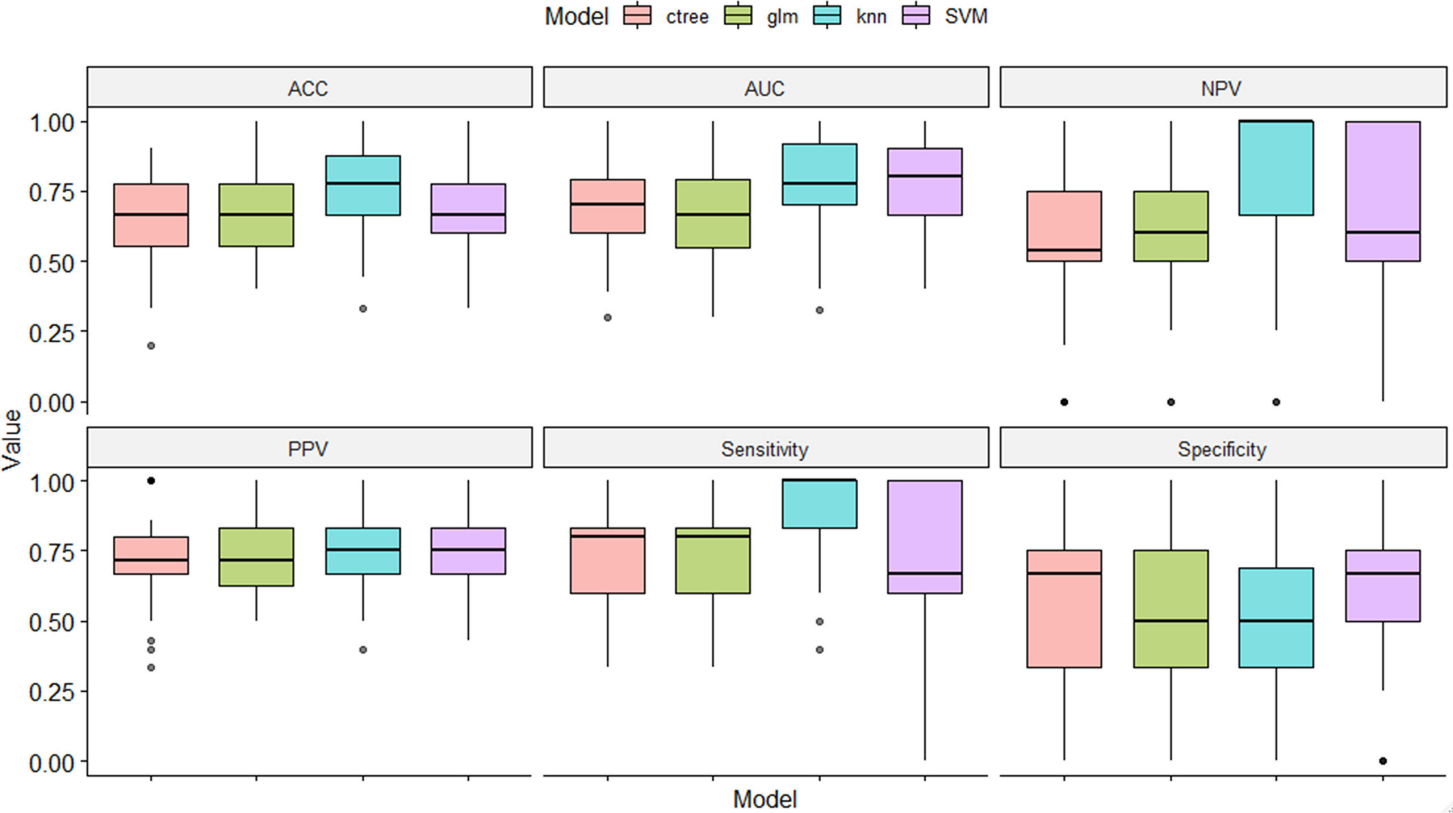
Performance comparison of the four classification machine learning models in distinguishing benign from malignant masses.

In the training set, 13 top-performing features (histogram, shape, and texture features), including 5 and 8 from the CC and MLO views, respectively, were finally selected by the LASSO logistic regression model (**Figures 2A, B**). **Figure 2C** shows the selected radiomics features, and the MLO view had more features than the CC view (8 and 5, respectively). Four classification machine learning models were constructed using the above selected 13 top-performing features, and the performances of the four classification machine learning models are shown in **Figure 3**. The logistic regression model had high AUC values of 0.91 for the training set (**Figure 3A**) and 0.87 for the test set (**Figure 3B**), but the LASSO-based machine learning model showed the best detection performance. The boxplot in **Figure 4** shows the accuracies, AUCs, NPV, PPV, and sensitivities and specificities of the four models after a 100-time cross-validation.

**Figure 2D** shows the rad-score of each patient determined by logistic regression. Individuals with cancerous tumors generally had higher rad-scores compared to the benign group, and rad-scores were statistically different between individuals with benign and malignant masses in the training and test cohorts (both *P*<0.001). The generated radiomics signature had good predictive accuracy, with AUCs of 0.91 (95% CI 0.86–0.98) and 0.87 (95%CI 0.76–0.99) in the training and test sets, respectively (**Figure 2E**).

### Nomogram

**Figure 5:**
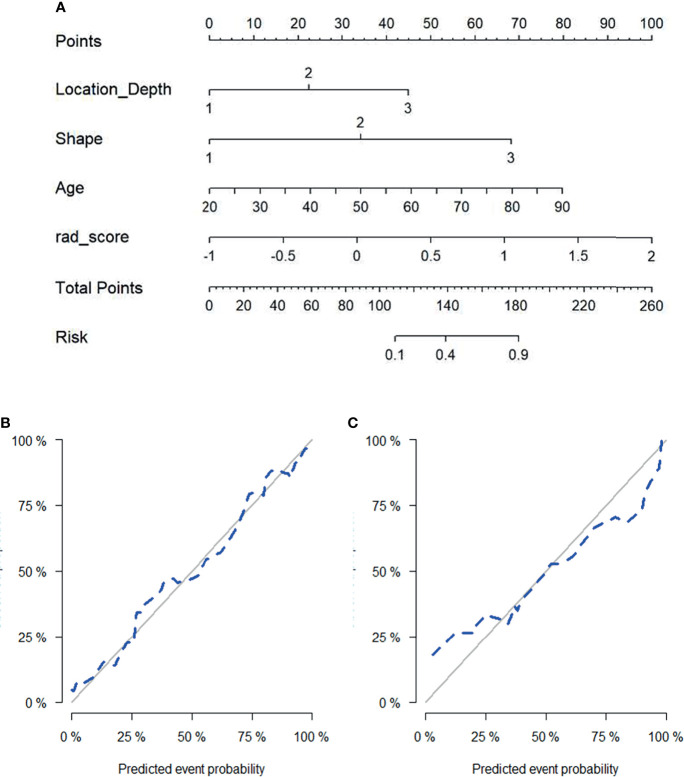
Radiomics nomogram for predicting malignant status of round-like tumors **(A)**. Calibration curves of the radiomics nomogram in the training set **(B)** and test set **(C)**.

According to multivariate logistic regression, location (depth), shape (US), age, and the radiomics signature all independently predicted malignancy in round-like tumors and were therefore included in a radiomics nomogram (**Figure 5A**). **Figures 5B, C** depict the nomogram’s calibration curves. In both the training and test cohorts, the curves reflected good calibration, and the Hosmer-Lemeshow test showed non-significance (*P*=0.375), (**Figures 5B, C**).

**Figure 6:**
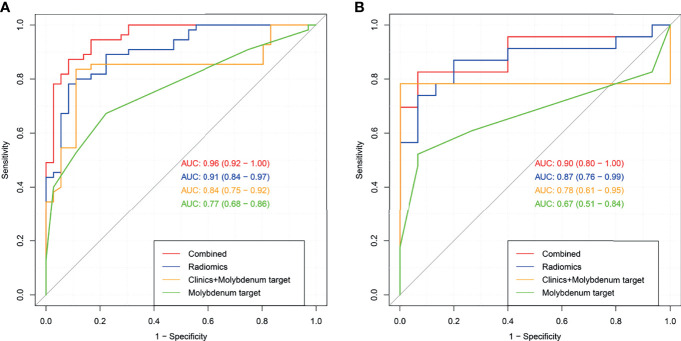
Receiver operating characteristic (ROC) curves for digital mammography, the clinical model, the radiomics signature and the radiomics nomogram in the training set **(A)** and test set **(B)**.

**Figures 6A, B** both present four ROC curves that compare digital mammography, the clinical model, the radiomics signature, and the radiomics nomogram for efficiency in differentiating round-like masses. The DeLong’s test showed that the ROC curves of the radiomics nomogram and digital mammography were statistically different (*P*<0.001), and the radiomics nomogram had an AUC of 0.90 (95% CI 0.80-1.00) in the test set, suggesting a significantly higher performance versus the prediction model constructed only with digital mammography features, which had an AUC of 0.67 (95% CI 0.51-0.84) in the test cohort.

The radiomics nomogram had high efficacy in detecting malignancy, with accuracy, sensitivity, specificity, PPV, and NPV of 0.890, 0.941, 0.825, 0.873,and 0.917, respectively, in the training cohort, and 0.868, 0.950, 0.778, 0.826, and 0.933 in the test cohort, respectively (**Table 5**). Machine learning-based mammography radiomics had an AUC of 0.87 (95% CI 0.76-0.99), indicating a better performance than the clinical model (AUC=0.78, 95% CI 0.61-0.95 in the test cohort) (**Figure 6B**). In the test set, the radiomics signature had higher specificity and PPV compared to the radiomics nomogram, and the radiomics nomogram had improved predictive ability (accuracy, sensitivity, and NPV) compared to the radiomics signature in distinguishing benign and malignant round-like tumors (**Table 5**).

**Table 5:** Performances of the predictive models in distinguishing benign from malignant tumors.

	Model	Accuracy	Sensitivity	Specificity	PPV	NPV
Training	DM	0.670 (0.564-0.765)	0.879	0.552	0.527	0.889
	Clinics	0.791 (0.693-0.869)	0.909	0.681	0.727	0.888
	Radiomics	0.835 (0.743-0.905)	0.782	0.917	0.935	0.733
	Combined	0.890 (0.807-0.946)	0.941	0.825	0.873	0.917
Test	DM	0.684 (0.513-0.825)	0. 923	0.56	0.522	0.933
	Clinics	0.711 (0.541-0.846)	1	0.577	0.522	1
	Radiomics	0.789 (0.627-0.904)	0.696	0.933	0.941	0.667
	Combined	0.868 (0.719-0.956)	0.95	0.778	0.826	0.933

DM, digital mammography; PPV, positive predictive value; NPV, negative predictive value.

## Discussion

This study developed a radiomics signature for predicting malignancy in round-like masses that had good accuracy in identifying the type of lesions (AUC=0.87 in the entire population). Further, a novel radiomics nomogram, built by utilizing multivariate logistic regression data, showed good calibration and was able to distinguish benign from malignant tumors in both the training and test data sets. The AUC of this signature was 0.90, suggesting a higher predictive value of the nomogram compared to mammography alone (AUC=0.67) as well as to the clinical model (AUC=0.78) that was established on the basis of age, DM, and US. In clinic DM and US characteristics are relied upon routinely for differential diagnosis. However, their values are dependent upon the radiologist**’**s experience. In addition, radiomics features are purely objective and quantitative.

This study compared four commonly used classification machine learning methods (SVM, C-Tree, k-NN, and logistic analysis) and found that LASSO had the best performance. As shown above, LASSO had higher AUC compared with the remaining classification machine learning methods. Indeed, LASSO can perform both feature selection and normalization for improving prediction accuracy (16) and is able to combine selected radiomic parameters for generating a radiomic signature (17, 18).

Additionally, the radiomics model had a higher ratio of features based on the MLO view compared with the CC view in this study, consistent with one previous study (19), suggesting that the MLO view might be more informative than its CC counterpart. Of course, combining both views provides more data as compared to each individual view (19). The radiomics features selected for modeling in this study included first-order, shape, and texture (including GLCM, GLSZM, and GLRLM) features. Texture features accounted for the largest proportion (7/13), and their correlation coefficients were relatively larger than other features as well. This also indicates that radiomics can reveal deep internal features.

Large Area Low Gray Level Emphasis (LALGLE) assesses the joint distribution of larger size zones showing lower gray-level values in a tumor image. In this study, both the original extraction technique and wavelet analysis were able to extract features from the oblique MLO and CC views. The feature weight was large, and two of three features had the highest magnitude of correlation coefficients (-0.556 and -0.48) in the feature set. Therefore, these features were negatively correlated with malignant status, which may be explained by the fact that most malignant masses have relatively dense cells and elevated density.

Size Zone Nonuniformity Normalized (SZNN) assesses size zone volume variability on a whole image, with reduced values suggesting elevated homogeneity among zone size volumes. The correlation coefficient here (0.334) was relatively large in the feature set, and positively correlated with malignant status, indicating high heterogeneity of malignant lesions. Furthermore, autocorrelation reflects the magnitude of texture fineness and coarseness, and in this study, this latter feature was positively correlated with malignant status, with a correlation coefficient of 0.133. The texture of malignant masses is generally coarser than that of benign counterparts. Compared with most irregular malignant masses, round-like masses show relatively more uniform growth rates and finer texture, which may explain the lower correlation coefficient.

Five first-order features were also selected in this study, and most of them had low correlation coefficients. Only MLO_wavelet-HHL_firstorder_Mean had a high correlation coefficient (0.329), indicating that the feature was positively correlated with malignant status, which can be explained by the elevated density of malignant tumor cells. Comparing the tumor and contralateral breast gland density by univariate analysis of clinical characteristics, significant differences were found between benign and malignant masses as well.

Multiple studies have shown that radiomics can provide valuable information for clinical diagnostic and prognostic assessments (20–26), and previous researchers have already evaluated DM-derived radiomics for categorizing microcalcification (27), tumors (28), and breast cancer by molecular properties (19, 29). This study focused on the masses that are most difficult to assign to the malignant and benign groups by DM. Consistent with the literature (12), jointly applying DM and radiomics was able to increase overall diagnostic performance remarkably. Such a combination can be used to examine tumor heterogeneity more comprehensively and quantitatively when compared to morphological visual assessment alone.

However, this study is not without its limitations. First, diseased and normal tissues show no overt boundaries in DM, and ROIs were not automatically generated. Therefore, irregularities resulting from manual selection were inevitable. Second, since the sample sizes of cases with specific histopathological subtypes of breast cancer were small, their differential diagnoses by radiomics could not be performed. Further research is therefore needed to address this issue. Finally, our results require multicenter verification with large trials in order to generate more evidence for clinical application.

## Conclusions

This study revealed that DM-based radiomics has good performance in distinguishing benign from malignant round-like masses, and the first-of-its-kind radiomics nomogram was developed and validated for such discrimination, achieving good accuracy. Indeed, DM-derived radiomics has an important clinical value in providing quantitative data to help clinicians read and interpret mammograms.

## Data Availability Statement

The datasets examined in this study are available from the corresponding authors upon reasonable request.

## Ethics Statement

The Institutional Review Board of Minhang Hospital, Shanghai, China, approved the current retrospective trial and did not require signed informed consent.

## Author Contributions

LW, YD, BZ, and BS conceived and designed the study. WY, HW, JS, WL, JX, RW, WH, and YG performed the experiments and collected the data. LW and YD wrote the first draft of the manuscript. All authors read and approved the final manuscript.

## Funding

The current research was financially supported by the Science and Technology Commission of Minhang District, Shanghai (grant number: 2019MHZ018).

## Conflict of Interest

Author YG was employed by General Electric (GE) Healthcare.

The remaining authors declare that the research was conducted in the absence of any commercial or financial relationships that could be construed as a potential conflict of interest.

## Publisher’s Note

All claims expressed in this article are solely those of the authors and do not necessarily represent those of their affiliated organizations, or those of the publisher, the editors and the reviewers. Any product that may be evaluated in this article, or claim that may be made by its manufacturer, is not guaranteed or endorsed by the publisher.

## Abbreviations

DM, Digital mammography; BI-RADS, Breast Imaging Reporting and Data System; US, ultrasound; MRI, magnetic resonance imaging; DCE-MRI, dynamic contrast-enhanced MRI; PACS, Picture Archiving and Communication System; CC, cranial caudal; MLO, mediolateral oblique; DICOM, Digital Imaging and Communications in Medicine; ROI, Region of interest; ICC, intraclass correlation coefficient; CI, confidence interval; mRMR, maximum correlation minimum redundancy; LASSO, least absolute shrinkage and selection operator; SVM, support vector machine; k-NN, k-Nearest Neighbor; ROC, receiver operating characteristic; AUC, area under the receiver operating characteristic curve; DCA, Decision curve analysis; PPV, positive predictive value; NPV, negative predictive value.
